# A protocol for culturing environmental strains of the Buruli ulcer agent, *Mycobacterium ulcerans*

**DOI:** 10.1038/s41598-018-25278-y

**Published:** 2018-04-30

**Authors:** Dezemon Zingue, Arup Panda, Michel Drancourt

**Affiliations:** 1 Aix Marseille Université, URMITE, UMR CNRS 7278, IRD 198, INSERM 1095, IHU Méditerranée Infection, Marseille, 13005 France; 2 Aix Marseille Université, MEPHI, IRD, IHU Méditerranée Infection, Marseille, 13005 France; 3 Aix Marseille Université, CNRS, Centrale Marseille, I2M UMR 7373, Equipe Biologique et Modélisation, Marseille, France

## Abstract

Contaminations and fastidiousness of *M. ulcerans* may have both hamper isolation of strains from environmental sources. We aimed to optimize decontamination and culture of environmental samples to circumvent both limitations. Three strains of *M. ulcerans* cultured onto Middlebrook 7H10 at 30 °C for 20 days yielded a significantly higher number of colonies in micro-aerophilic atmosphere compared to ambient atmosphere, 5% CO_2_ and anaerobic atmosphere. In a second step, we observed that *M. ulcerans* genome uniquely encoded chitinase, fucosidase and A-D-GlcNAc-diphosphoryl polyprenol A-3-L-rhamnosyl transferase giving *M. ulcerans* the potential to metabolize chitine, fucose and N-acetyl galactosamine (NAG), respectively. A significant growth-promoting effect of 0.2 mg/mL chitin (p < 0.05), 0.01 mg/mL N-acetyl galactosamine (p < 0.05), 0.01 mg/mL fucose (p < 0.05) was observed with *M. ulcerans* indicating that NAG alone or combined with fucose and chitin could complement Middlebrook 7H10. Finally, the protocol combining 1% chlorhexidine decontamination with micro-aerophilic incubation on Middlebrook 7H10 medium containing chitin (0.2%), NAG (0.01%) and fucose (0.01%) medium and auto-fluorescence detection of colonies allowed for the isolation of one mycolactone-encoding strain from *Thryonomys swinderianus* (aulacode) feces specimens collected near the Kossou Dam, C*ô*te d’Ivoire. We propose that incubation of chlorhexidine-decontaminated environmental specimens on Middlebrook 7H10-enriched medium under micro-aerophilic atmosphere at 30 °C may be used for the tentative isolation of *M. ulcerans* strains from potential environmental sources.

## Introduction

Buruli ulcer is a World Health Organization (WHO)-notifiable, yet neglected infection of the cutaneous and subcutaneous tissues caused by the nontuberculous *Mycobacterium ulcerans*^1^. This pathogen emerged from a common ancestor with the environmental *Mycobacterium marinum*, after genomic reduction and the acquisition of a 174-kb pMUM001 plasmid encoding a macrolide mycolactone toxin, the major virulence factor for *M. ulcerans*^2–4^. *M. ulcerans* was initially isolated from sub-cutaneous lesions in patients in Bairnsdale, Australia, where Buruli ulcer (Bairnsdale ulcer) was initially described^5^. For more than 70 years, Buruli ulcer cases have been notified in patients residing in 33 countries mainly in the rural and tropical regions of Africa, significantly less in South America, in addition to Australia and Japan^1^. For an example, in 2014, 2,200 new cases were notified by 12 countries and most of the patients were children under 15 years^1^. The laboratory diagnostic of Buruli ulcer is made by microscopy, histopathology and PCR-based detection of *M*. *ulcerans*-specific sequences, including the IS2404, IS2606 and ketoreductase-B domain of the mycolactone polyketide synthase genes^1,6–8^. Successful isolation and culture of *M. ulcerans* from clinical lesions depends on several parameters, including the exact sampled site (most bacilli are in the deepest areas of the skin), or the type of decontamination method or culture medium and culture conditions used^8–11^. Whilst *M. ulcerans* grows on similar culture media as *Mycobacterium tuberculosis*, i.e. on Löwenstein-Jensen medium, Brown and Buckle or Ogawa medium, microaerophilic atmosphere and optimal temperature of 28–33 °C are required for this pathogen^8–11^. In a clinical diagnostic laboratory, primary cultures are usually positive within a 6–12-week incubation, but a much longer incubation period of up to nine months may be necessary to obtain isolates, illustrating the fastidiousness of this microbe^11,12^.

The fact that thousands *M. ulcerans* isolates have been made from clinical sources sharply contrasts with the fact that several attempts to culture *M. ulcerans* from many specimens of flora and fauna remained unsuccessful^13,14^. However, numerous PCR-based investigations indicated that potential reservoirs or host carriers were localized in aquatic environments where *M. ulcerans* may be able to colonize different ecological niches eventually scattered along a food chain^15–19^. Culture of *M. ulcerans* from environmental samples is tedious and understanding the ecology of *M. ulcerans* has been severely hampered by the extreme difficulty of culturing the organism directly from the environment^14,20,21^. Cultures of collected diverse samples (water, soil, fish, rodents, biting flies, reptiles) from Buruli ulcer endemic areas failed to yield *M. ulcerans* a long time ago^13^, though testing of samples by molecular biology found *M. ulcerans* DNA^7,22–30^. Finally, only one environmental *M. ulcerans* (*M. ulcerans* 00–1441 from a Buruli ulcer endemic area in Benin, West Africa) isolate has been firmly confirmed on Löwenstein-Jensen medium after 15-day of incubation in BACTEC 12b broth and three successive passages in mouse footpad P1, P2 and P3 for nine months, six months and 12 months, followed by culture on Löwenstein-Jensen for two months^21^. Three additional reported strains included two IS2404-PCR positive strains from two samples of aquatic plants and two wild aquatic insects collected in a Buruli ulcer endemic area of Côte d’Ivoire^31,32^ and two *M. ulcerans* strains from moss and soil in Ghana^33^. None of these strains have been deposited in public collection.

The fact that only a few environmental isolates have been made after such a long experiment, suggests that contamination by fast-growing bacteria or mycobacteria and fungi of the environmental samples along with poorly appropriate culture media limited the isolation of *M. ulcerans* from environmental sources. Also when contaminants are present in the sample, they limit and inhibit the growth of *M. ulcerans*. Contaminants not eliminated by the decontamination method eventually rot or transferring the culture medium rendering impossible the incubation of cultures on a long period necessary for the isolation of *M. ulcerans*. There is thus a need to develop new protocols and innovative media to improve the recovery of *M. ulcerans* in primary culture from environmental sources in order to assess the viability of the pathogen in these sources^34^. The availability of the complete genome sequence of *M. ulcerans* is a boon to better known metabolic activities which could support the development of innovative culture media, as previously reported for some other fastidious pathogens^35^.

In the perspective of achieving a culture-based field investigation of *M. ulcerans*, we aimed at improving the decontamination of samples along with the composition of culture media in order to optimize the chance of recovering additional environmental *M. ulcerans* isolates.

## Results

### Effect of atmosphere in culturing *M. ulcerans* onto Middlebrook 7H10

**Figure 1:**
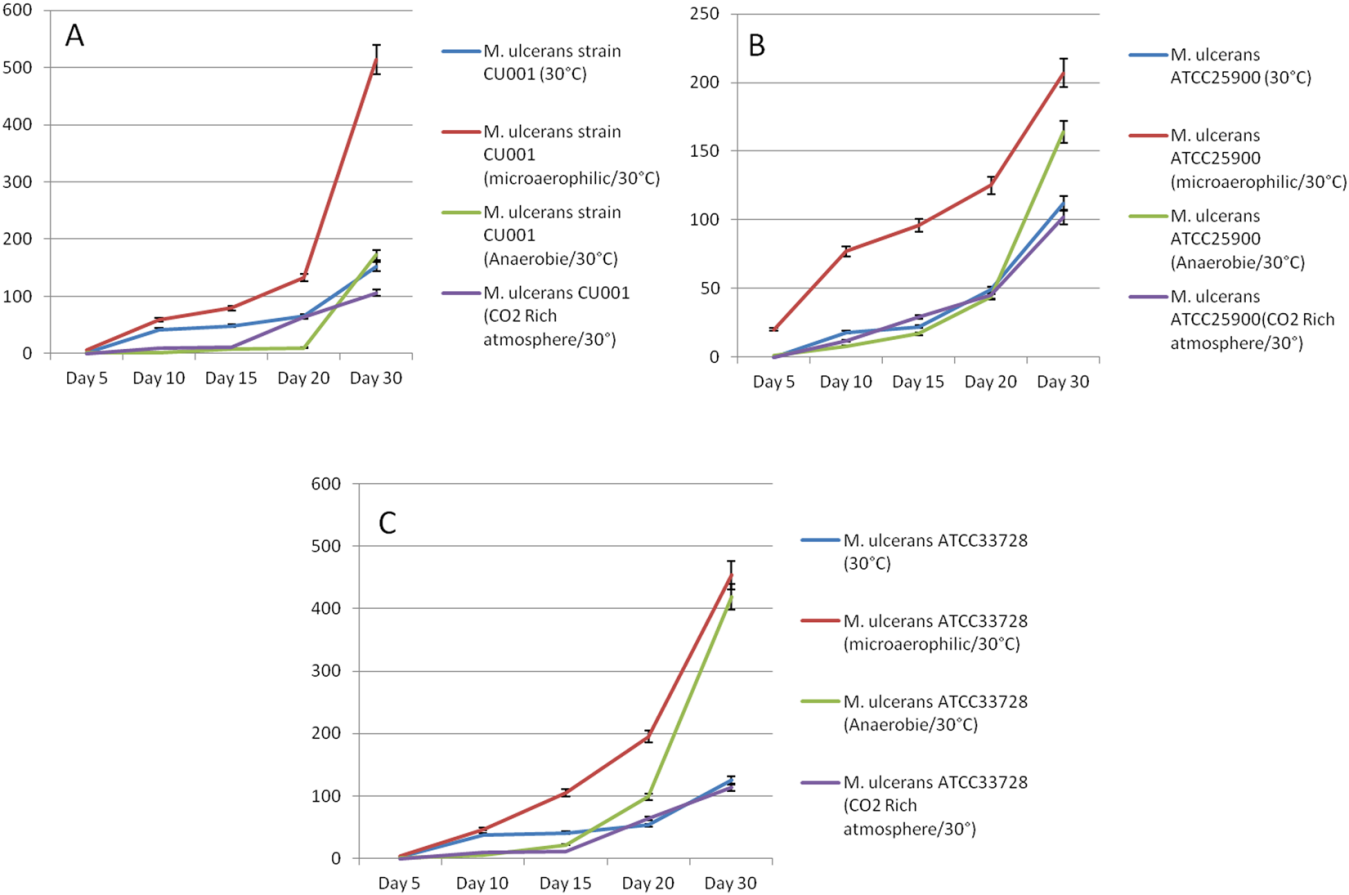
Triplicate culture of 10^4^ bacilli of *M. ulcerans* CU001 (**A**), *M. ulcerans* ATCC25900 (**B**) and *M. ulcerans* ATCC33728 (**C**) in Middlebrook 7H10 under different atmospheres (ambient atmosphere, and microaerophilic, anaerobic and 5% CO_2_-enriched atmosphere). Microaerophilic atmosphere yielded the optimal growth of *M. ulcerans* (red line).

We observed an enhanced growth of *M. ulcerans* under micro-aerophilic atmosphere at day 20 post-incubation on Middlebrook 7H10 medium at 30 °C. The number of colonies of *M. ulcerans* CU001 was significantly higher in micro-aerophilic atmosphere (133 ± 6 CFUs) than under ambient atmosphere (65 ± 14 CFUs; p = 0.002) or 5% CO_2_ atmosphere (36 ± 14 CFUs; p = 0.0001) or anaerobic atmosphere (10 ± 5 CFUs; p = 0.0001); likewise, *M. ulcerans* ATCC25900 yielded 125 ± 18 CFUs in micro-aerophilic atmosphere compared to 49 ± 1.7 CFUs (p = 0.002) under ambient atmosphere, 45 ± 11 CFUs (p = 0.003) under 5% CO_2_ atmosphere and (44 ± 7 CFUs; p = 0.002) in anaerobic atmosphere; and *M. ulcerans* ATCC33728 yielded 195 ± 13 CFUs under micro-aerophilic atmosphere compared to 53 ± 6 CFUs (p = 0.0001) in ambient atmosphere, 64 ± 32 CFUs (p = 0.003) under 5% CO2 atmosphere and 99 ± 11 CFUs; p = 0.001 in anaerobic atmosphere (Fig. 1).

Cultures at 37 °C. remained negative during the same period of incubation.

### Effect of growth promoters on culturing *M. ulcerans* strains

We incorporated chitin, fucose and N-acetylgalactosamine into Middlebrook 7H10 medium in order to experimentally test their growth-promoting effect on *M. ulcerans*. The experimental data were authenticated by the negativity of the negative controls used in every experiment and the reproducibility of data over three different *M. ulcerans* strains and three independent experiments.

**Figure 2:**
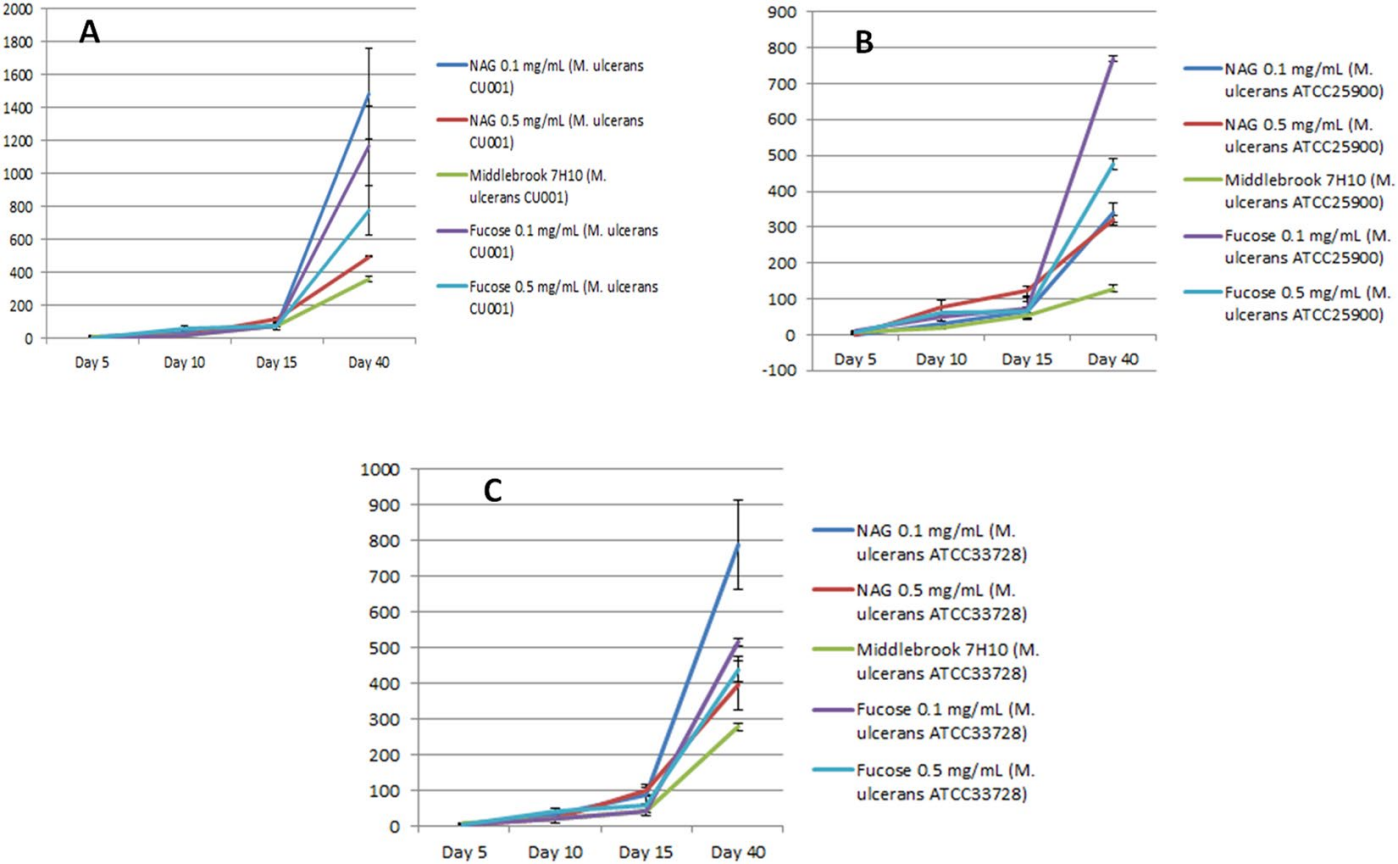
Growth of *M. ulcerans* (10^4^ bacilli) onto Middlebrook 7H10 enriched with high concentration of N-acethyl galactosamine (0.1 mg/mL, 0.5 mg/mL) and fucose (0.1 mg/mL, 0.5 (**A**). *M. ulcerans* CU001; (**B**) *M. ulcerans* ATCC25900; (**C**) *M. ulcerans* ATCC33728.

Incorporation of 0.1 mg/mL fucose or 0.1 mg/mL N-acetyl galactosamine into Middlebrook 7H10 base yielded no significant difference in the growth of *M. ulcerans* until day 15. However, from day 15 to day 40, the number of colonies was significantly higher on Middlebrook 7H10 medium enriched with 0.1 mg/mL N-acetyl galactosamine (1,485 ± 275 CFUs for *M. ulcerans* CU001, 340 ± 28 CFUs for *M. ulcerans* ATCC25900, 788 ± 125 CFUs for *M. ulcerans* ATCC33728) or Middlebrook 7H10 enriched with 0.1 mg/mL fucose (1,770 ± 241 CFUs for *M. ulcerans* CU001, 770 ± 9 CFUs for *M. ulcerans* ATCC25900, 516 ± 11 CFUs for *M. ulcerans* ATCC33728) than in standard Middlebrook 7H10 medium (359 ± 16 CFUs for *M. ulcerans* CU001, 129 ± 8 CFUs for *M. ulcerans* ATCC25900, 278 ± 9 CFUs for *M. ulcerans* ATCC33728) (p < 0.05) (Fig. 2).

**Figure 3:**
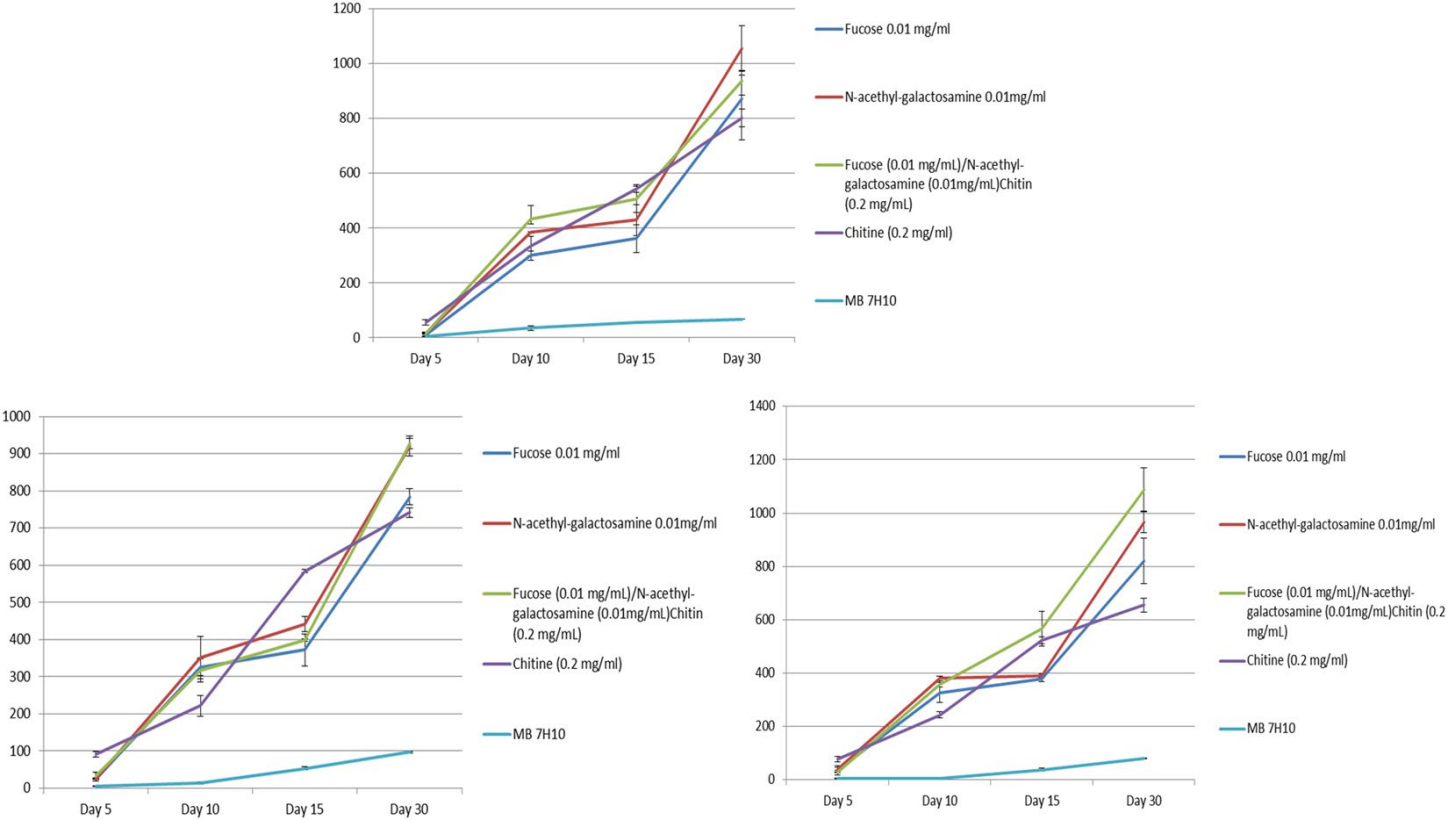
Improved growth of *M. ulcerans* strains (10^4^ bacilli) by N-acetyl galactosamine (NAG) (0.01 mg/mL), fucose (0.01 mg/mL), chitin (0.2 mg/mL) and DZ medium (NAG (0.01 mg/mL), fucose (0.01 mg/mL), chitin (0.2 mg/mL) versus growth onto Middlebrook 7H10 (p < 0.05). (**A**) *M. ulcerans* CU001; (**B**) *M. ulcerans* ATCC25900; (**C**) *M. ulcerans* ATCC33728.

Furthermore, the number of colonies was significantly higher on Middlebrook 7H10 medium enriched with 0.01 mg/mL N-acetyl galactosamine (1,054 ± 84 CFUs for *M. ulcerans* CU001, 920 ± 7 CFUs for *M. ulceran s* ATCC25900, 967 ± 40 CFUs for *M. ulcerans* ATCC33728) or Middlebrook 7H10 enriched with 0.01 mg/mL fucose (871 ± 102 CFUs for *M. ulcerans* CU001, 784 ± 53 CFUs for *M. ulcerans* ATCC25900, 821 ± 56 CFUs for *M. ulcerans* ATCC33728) or Middlebrook 7H10 enriched with 0.2 mg/mL chitin (802 ± 82 CFUs for *M. ulcerans* CU001, 741 ± 42 CFUs for *M. ulcerans* ATCC25900, 655 ± 25 CFUs for *M. ulcerans* ATCC33728) than in standard Middlebrook 7H10 medium (67 ± 1 CFUs for *M. ulcerans* CU001, 97 ± 2 CFUs for *M. ulcerans* ATCC25900, 80 ± 2 CFUs for *M. ulcerans* ATCC33728) (p < 0.05) (Fig. 3).

The number of colonies was significantly higher on Middlebrook 7H10 medium enriched with a mix of the three growth promoters called DZ medium (0.01 mg/mL N-acetyl galactosamine, 0.01 mg/mL fucose and 0.2 mg/mL chitin) (936 ± 21 CFUs for *M. ulcerans* CU001, 927 ± 57 CFUs for *M. ulcerans* ATCC25900, 1087 ± 82 CFUs for *M. ulcerans* ATCC33728) than in standard Middlebrook 7H10 medium (67 ± 1 CFUs for *M. ulcerans* CU001, 97 ± 2 CFUs for *M. ulcerans* ATCC25900, 80 ± 2 CFUs for *M. ulcerans* ATCC33728) (p < 0.05) (Fig. 3). The doubling time of *M. ulcerans* during the exponential growth was measured at 1.65 ± 0.01 days with chitin, 1.74 ± 0.03 days with N-acetyl-galactosamine, 1.75 ± 0.01 days for fucose, 1.68 ± 0.05 days with chitin/N-acetyl-galactosamine/fucose and 2.40 ± 0.58 days with the control Middlebrook 7H10 medium.

### Chlorhexidine decontamination of river freshwater artificially inoculated with *M. ulcerans* strains

Direct seeding without decontamination of river freshwater sample onto 5% sheep-blood Columbia agar combined with MALDI-TOF-MS identification of colonies yielded *Acinetobacter baumannii, Acinetobacter baylyi, Acinetobacter junii, Acinetobacter nosocomialis, Aeromonas caviae, Aeromonas ichthiosmia, Aeromonas hydrophila, Aeromonas veronii, Brevibacterium luteolum, Brevibacterium paucivorans, Escherichia coli, Klebsiella pneumoniae* and *Serratia marcescens* within 48 hours of incubation.

**Figure 4:**
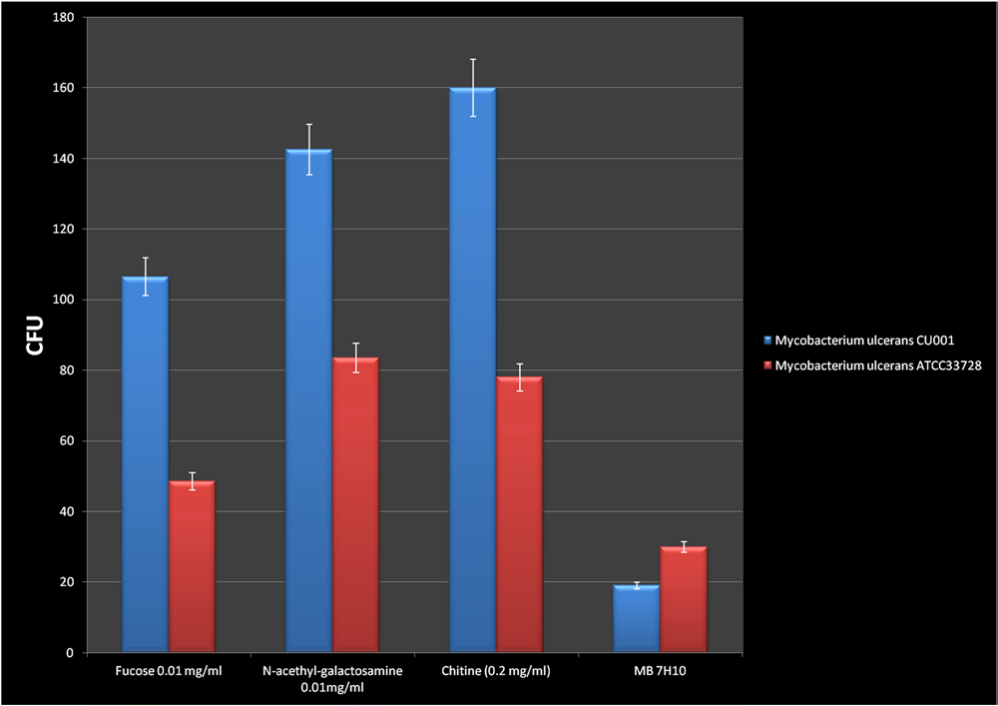
Growth count at day 30 of 10^4^ bacilli of *M. ulcerans* CU001 and *M. ulcerans* ATCC33728 colonies after 1% chlorhexidine decontamination of river freshwater artificially inoculated with *M. ulcerans* strains and cultured onto growth promoters and Middlebrook 7H10. Blue column: *Mycobacterium ulcerans* CU001; Red column: *Mycobacterium ulcerans* ATCC33728.

After 1% chlorhexidine decontamination of freshwater sample, the culture onto sheep-blood Columbia agar remained sterile without contamination. The same freshwater sample mocked- inoculated with *M. ulcerans* strain (CU001 and ATCC33728 separately) and chlorhexidine-decontaminated yielded autofluorescent colonies of *M. ulcerans*, starting at day 15 after inoculation onto the chitin, fucose and NAG growth promoters media. Colony counting was done on day 30 (Fig. 4). Several colonies obtained during this step were confirmed by RT-PCR targeting specific genomic regions of *M. ulcerans* (IS2404, IS2606, KR-B) in order to confirm *M. ulcerans* colonies. RT-PCR results where positive for all IS2404, IS2606 and KR-B targets.

### Detecting IS 2404, IS 2606 and KR-B targets in environment samples, Côte d’Ivoire

While the negative controls remained negative and all the samples were free of PCR inhibition, 5/12 water without plant debris (41.66%) were positive for KR-B gene, insertion sequence IS2404 and IS2606; five (41.66%) were positive for KR-B and IS2606; one was positive for IS2404 and IS2606 and one was negative. Among the eight water with plants debris, two (25%) were positive for KR-B and IS2404; one was positive for IS2404 and IS2606; one was positive for IS2404 and one was positive for IS2606; and three were negative. No *Thryonomys swinderianus’s* aulacode feces were definitely found positive according to our criteria while one was positive for IS2404 and IS2606, one was positive for IS2404, one was positive for IS2606 and eight were negative (Table 1). Further, the calculated values for Δ*Ct* (IS2606 - IS2404) from PCR-positive environmental samples were ≤ 3.32 (95% CI = 0.43–1.70). These low values suggest that environmental mycobacteria here detected to harbor the two IS2606 and IS 2404 insertion sequences, are not *M. ulcerans* which is acknowledged to harbor two orders of magnitude more copies of IS 2404 than IS2606 [ref.^7^].

**Table 1:** Real-time PCR (RT-PCR) results of *M. ulcerans* DNA detection in aulacode feces (FAG), water (EAU) and water containing plant debris (VEG) by using detection of KR-B gene and insertion sequences IS2404 and IS2606.

Samples	KR-B	IS2404	IS*2606*
KON-FAG 1			+29.65
KON-FAG 2			
KON-FAG 3			
KON-FAG 4		+33.04	+31.58
KON-FAG 5			
KON-FAG 6			
KON-FAG 7			
KON-FAG 8		+36.30	
KON-FAG 9			
KON-FAG 10			
KON-FAG 11			
KON-EAU1	+33.11	+30.97	+29.85
KON-EAU 2	+32.14	+32.00	+30.77
KON-EAU 3			
KON-EAU 4	+31.56		+29.94
KON-EAU 5	+31.36		+29.64
KON-EAU 6	+32.74		+30.64
KON-EAU 7	+30.67		+29.18
KON-EAU 8	+30.15		+27.99
KON-EAU 9		+30.15	+30.08
KON-EAU 10	+32.75	+32.55	+31.75
KON-EAU 11	+32.22	+31.58	+30.51
KON-EAU 12	+32.37	+32.12	+29.85
KON-VEG 1			+30.97
KON-VEG 2		+32.85	
KON-VEG 3			
KON-VEG 4	+34.70		+30.09
KON-VEG 5			
KON-VEG 6		+32.34	+31.64
KON-VEG 7			
KON-VEG 8	+32.23		+29.07

Ct values are indicated for specimens detected as positive.

### Culturing *M. ulcerans* micro-colony in environment samples, Côte d’Ivoire

**Figure 5:**
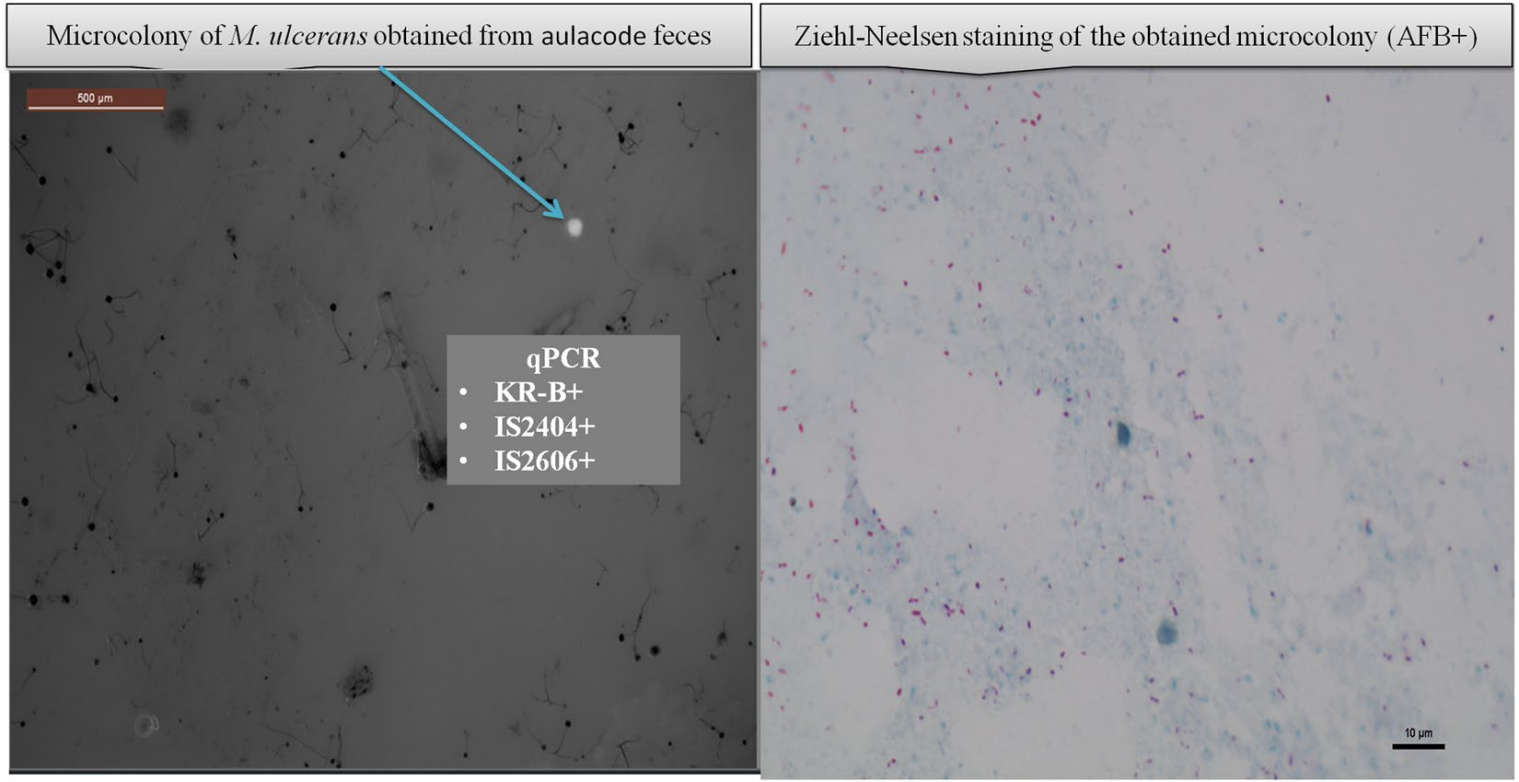
Culturing one aulacode feces sample collected in Côte d**’**Ivoire on Middlebrook 7H10 enriched with growth promoters yielded one micro-colony observed by autofluorescence (arrow) (left panel). Right panel exhibits Ziehl-Neelsen staining of the micro colony further identified as *M. ulcerans* by positive RT-qPCR for KR-B gene, IS*2404* and IS2606.

The culture of the 31 samples decontaminated with 1% chlorhexidine yielded one auto-fluorescent micro-colony isolated from one IS2404-positive aulacode feces sample after 45-day incubation on the DZ medium at 30 °C under a micro-aerophilic atmosphere condition. This unique micro-colony yielded Ziehl-Neelsen -positive bacilli identified as a MPM after the RT-PCR positivity for KR-B, IS2404 and IS2606 in the presence of negative controls (Fig. 5). However, calculated value for Δ*Ct* (IS2606-IS2404) of 2. 97 was not in favor that the colony was of *M. ulcerans*, but rather in favor of other mycobacteria carrying the three detected molecular targets^7^. Sub-culturing onto the DZ medium of the micro-colony material remaining after identification failed.

## Discussion

We here report on a culture protocol for the tentative recovery of viable *M. ulcerans* mycobacteria from environmental sources.

Temperature of incubation is a crucial point for the culture of mycobacteria as previously reported^36^. Indeed, *M. ulcerans* strains have optimal growth between 28–32 °C; they are very sensitive to higher temperatures, a temperature of 41 °C over a period of 24 hours kills more than 90% of the bacilli^13^. This observation gives indications for the storage of environmental samples for *M. ulcerans* culture in endemic countries where the ambient temperature may reach values in-between 37 °C–45 °C, suggesting that samples should be stored at lower temperatures into any appropriate transport medium before inoculation. We then observed that growth of the *M. ulcerans* strains here investigated was significantly more rapid in micro-aerophilic atmosphere than in ambient atmosphere and in a 5% CO_2_-enriched atmosphere. It was previously suggested that *M. ulcerans* is capable of growth under aerobic but not anaerobic conditions^3^ and to survive anaerobic conditions^37^. During our experiment we observed a significant growth of the three strains of *M. ulcerans* cultured under anaerobic conditions after day 20 of incubation. Indeed, breaking anaerobic atmosphere may occur during which a suitable atmospheric condition for the growth of mycobacteria was created during colonies count and/or pocket replacement every five days. Further, a possible activity of the *cydA* locus in *M. ulcerans* may sustain the ability of this strains to survive under low-oxygen conditions^37^.

To develop an innovative culture medium for improve tentative isolation and growth of *M. ulcerans*, we thought that comparative genome analysis reveal unique metabolic features and clues to enrich a Middlebrook 7H10 medium. Indeed, this approach has been successfully used to design a new culture medium for the fasditious pathogen *Tropheryma whipplei*, another Actinobacterium^35^. Accordingly, we incorporated chitin, fucose and N-acetylgalactosamine into a Middlebrook 7H10 medium in order to experimentally test their effect on the growth of *M. ulcerans*. The experimental data were authenticated by the negativity of the negative controls used in every experiment and the reproducibility of data over three different *M. ulcerans* strains culture and triplicate experiments. We observed that the N-acetyl-galactosamine-enriched Middlebrook 7H10 medium and three growth-promoters-Middlebrook 7H10 medium yielded similar results indicative that both media could be used for tentative isolation of MPM including *M. ulcerans* from environmental sources. Chitin is the second most abundant organic and renewable source in nature, after cellulose^38^. This linear homopolymer can be hydrolyzed at β-1,4-linkages by the enzymatic action of glycoside hydrolase enzymes, the chitinases (E.C. 3.2.1.14) and the N-acetylglucosaminidases (E.C. 3.2.1.52)^39,40^. Chitin is found in the structure of fungi, crustaceans (crabs, lobsters), insects, mollusks, cephalopods, fishes such as zebrafish (*Danio rerio*) and amphibians^41–43^. The derivatives of chitin play a crucial role in the interaction between higher plants and symbiotic bacteria; suggesting that chitin synthesis may serve roles other than the production of skeletal material^42^. N-acetyl galactosamine has been recognized as a minor covalently-bound amino sugar component of the cell wall of some slow-growing mycobacteria and orthologs of polyprenyl-phospho-N-acetyl-galactosaminyl synthase (ppgS), which are found in the genomes of slowly-growing mycobacteria including *M. bovis*, *M. bovis* BCG, *M. leprae*, *M. marinum* and *M. avium* subsp. *paratuberculosis*, as well as in *M. abscessus*; but not in the genomes of other rapidly growing *Mycobacterium* species such as *Mycobacterium smegmatis*^44,45^.

Chitinases are chitin-degrading enzymes belonging to the glycoside hydrolase family 18 (GH18) and 19 (GH19)^39^ (www.cazy.org). They act in a synergistic to perform the complete enzymatic hydrolysis of chitin to N-acetylglucosamine^46,47^. The GH18 family is widely distributed in all kingdoms, including viruses, bacteria, plants, fungi and animals^38^. Bacterial chitinases and chitin-binding proteins (CBPs) play a fundamental role in the degradation of the ubiquitous biopolymer chitin, and the degradation products serve as an important nutrient source for marine- and soil-dwelling bacteria^48–51^. *M. ulcerans* genomes encode for a GH18 compatible with a putative chitinase activity^3^. N-acetylglucosaminidases belong to glycoside hydrolase family 20 (GH20)^39^. α-l-Fucosidases are enzymes involved in metabolism of α-l-fucosylated molecules, compounds with a fundamental role in different life essential processes including development^52^. These enzymes play a fundamental role in the degradation of the ubiquitous biopolymer^39,46,50^. The degradation products serve as an important nutrient source for bacteria in the nature^51^. *M. ulcerans* may obtain energy and carbon from the degradation of plant saccharides which were demonstrated to stimulate *M. ulcerans* growth *in vitro*^14,53^. It was recently shown that chitin promoted growth of *M. ulcerans*^43^. Fucose was here tested after we observed that *M. ulcerans* contained an alpha-L-fucosidase cytoplasmic protein involved in carbohydrate transport and metabolism^39^.

It was proved that some green algae extracts stimulate the growth of *M. ulcerans*^14^. In a later study however, the growth of *M. ulcerans* was indirectly observed by using quantitative PCR kinetics, instead of the simple observation of growing colonies as reported here^43^.

The doubling time obtained with the culture of *M. ulcerans* strains onto each growth promoter was less than two days and was in agreement with previously reported values. The doubling time was estimated to be of approximately 36 h^54^, 1–2 days^55^ and 3.3 ± 0.56 days^14^. In the Dubos medium (a liquid medium), the doubling time was less than 48 hours at 33 °C^56^ and 44 hours in the Dubos medium without serum^57^. A doubling time estimated to be between three and four days has been reported^58^. In mice, the doubling time was approximately 3.5 days^59–61^. The discrepancy may be first due to the strain of *M. ulcerans*, secondly to the calculation method^57^ or the type of culture medium. In contrast, *M. marinum* has a doubling time of 6–11 hours^3^.

Culture onto 5% sheep-blood Columbia agar of freshwater sample after chlorhexidine decontamination remained sterile so, we deduced that the chlorhexidine decontamination method has been effective. Culture onto growth promoters’ media of chlorhexidine decontaminated freshwater sample mocked- inoculated with *M. ulcerans* strains allowed to isolate *M. ulcerans* colonies confirmed by RT-PCR.

Here, IS2404, IS2606 and KR-B molecular targets were detected by RT-PCR in water, plants debris and aulacodes feces, all collected in Côte d’Ivoire. The fact that the reproducible Ct values we obtained for IS2404 and IS2606 are not in line with those expected from *M. ulcerans* pure DNA, suggests these natural specimens do not contain purely *M. ulcerans* but rather other mycobacteria harboring these insertion sequences in variable numbers. *M. ulcerans’s* DNA has been detected previously in two feces from the aulacode collected in Côte d’Ivoire^22^ and in small mammal (*Mastomys*) in Ivory Coast^27^ suggesting that these animals may shelter and vehicle *M. ulcerans*.

Growth promoters allow the isolation of micro-colony which was positive for IS2404, IS2606 and KR-B. However, direct sub-culture onto the growth-promoters for more biological material failed. This failure could be explained by the very low inoculum remaining for the culture after we realized Zielh-Neelsen staining and the RT-PCR for the identification of the micro-colony. Our positive samples were collected in a Buruli ulcer endemic region in the centre of Côte d’Ivoire, closely related but different from a site where we previously PCR-amplified *M. ulcerans* DNA in the feces of aulacodes^22^. Therefore, this interesting yet limited observation warrants further field studies for confirmation.

## Conclusions

A 1% chlorhexidine decontamination and addition of appropriate concentration of N-acetyl galactosamine or combination of chitin, N-acetyl galactosamine and fucose to the standard Middlebrook 7H10 culture medium promoted the growth of *M. ulcerans* under microaerophilic atmosphere at 30 °C. These protocols are proposed as a first-line protocol for the tentative isolation of *M. ulcerans* strains during field campaigns in Buruli ulcer endemic areas.

## Methods

### Ethics statement

The study has been conducted with collection references strains of *M. ulcerans* and no experiment or test has been performed on patients or/and animals.

### *M. ulcerans* strains

Three strains of *M. ulcerans* isolated from different geographic origins were used throughout the study. *M. ulcerans* strain Cu001 (a gift from Prof Vincent Jarlier, Centre National de Référence des Mycobactéries, Paris France) was from Côte d’Ivoire^62^, *M. ulcerans* ATCC 33728 isolated from Japan and *M. ulcerans* ATCC 25900 belongs to the Borstel collection (Schröder 5392) and was probably isolated in Africa. The identification of these three strains were ensured by *rpoB* gene sequence analysis prior to experiments^63^. *M. ulcerans* strains were sub-cultured at 30 °C onto Middlebrook 7H10 agar medium supplemented with 10% (v/v) oleic acid/albumin/dextrose/catalase (OADC) (Becton Dickinson, Sparks, MD, USA) and 0.5% (v/v) glycerol (Sigma-Aldrich, Lyon, France) in a microaerophilic atmosphere until we have enough colonies to prepare inoculum. Then, a mycobacterial suspension was prepared by placing a loopful of colonies in a tube containing sterile phosphate buffered saline (PBS, pH 6.5) and sterile glass beads. The tube was vigorously vortexed in order to separate any bacterial aggregates and adjusted with PBS in setting the mycobacterial concentration of inoculum to 0.5 McFarland standards in order to obtain a final suspension containing 10^7^ acid-fast bacteria (AFB)/mL using a turbidimeter (Biolog Inc., Hayward, U.S.A). This suspension was shown to be free of clumps by microscopic examination after Ziehl-Neelsen staining. Then, a 10^6^ AFB/mL working suspension was prepared.

In all further experiments, colonies were observed and count by using a MZ-FLIII fluorescence microscope (Leica, Nanterre, France) equipped with a GFP filter and an ICA digital camera (Leica) to detect mycobacterial auto-fluorescence as previously described^64^. Counting of fluorescent colonies was performed using the Leica Application Suite software (Leica). The identification of colonies was confirmed by matrix assisted laser desorption ionization time of flight mass spectrometry (MALDI-TOF-MS) (Bruker Daltonics, Bremen, Germany) as previously described^65^.

### Testing atmosphere conditions for culturing *M. ulcerans*

The first step consisted in the culture of *M. ulcerans* under different conditions. For each *M. ulcerans* strain, a 100 µL-volume of a 10^5^ AFB/mL suspension corresponding to 10^4^ AFB was cultured in triplicate onto Middlebrook 7H10 medium supplemented with 10% (v/v) OADC and 0.5% (v/v) glycerol then, incubated at 30 °C under four different atmospheric conditions. The atmospheric growing conditions were artificially created by the use of sealed plastic pouch that can hold ten Middlebrook 7H10 medium poured in 55-mm diameter Petri dishes. The conditions of culture to produce standard, microaerophilic (5% oxygen), anaerobic (total absence of free oxygen and 8–14% CO_2_) and 5% CO_2_ rich atmosphere conditions with a 15% final concentration of oxygen, all in closes pouch were made respectively by simple culture method, microaerophilic condition using CampyGen Compact (OXOID Ltd, Basingstoke, Hampshire, England), Anaerobie Poche System/AnaeroGen Compact (OXOID Ltd, Basingstoke, Hants RG24 8PW, UK) and CO_2_ Rich atmosphere using CO_2_ Gen Compact (OXOID Ltd). All inoculated cultures and negative control inoculated with sterile PBS were incubated at 30 °C. In parallel, we cultured onto Middlebrook 7H10 medium supplemented with 10% (v/v) OADC and 0.5% (v/v) glycerol, the three strains of *M. ulcerans* (10^4^ AFB per Petrish dish), incubated at 37 °C. Cultures were all examined at day 5, day 10, day 15, day 20 and day 30 by a MZ-FLIII fluorescence microscope. Counting of fluorescent colonies was performed using the Leica Application Suite software.

### Growth-promoters

In a second step, we searched for genes encoding chitinase, N-acetyl galactosaminase (NAG) and fucosidase in completely sequenced *Mycobacterium* genomes (NCBI Gene Bank, last accessed in February 2016). To estimate the copy number of these genes, we considered protein functional description of the respective strains as documented in the gff files of NCBI Gene Bank bacterial genome repository. In each completely sequenced *Mycobacterium* genome, we counted the number of genes using the key terms “chitinase”, “cellulase”, “fucosidase” in their protein functional annotation column.

A total of 109 *Mycobacterium* genomes were screened in this analysis. The presence of one of these three genes was detected in 70 genomes and the presence of the three genes at once was detected only in the *M. ulcerans* genome.

Therefore, these three substances were tested as growth promoters for *M. ulcerans*. N-acetylgalactosamine (Sigma-Aldrich) and fucose (Sigma-Aldrich) were dissolved in sterile distilled water (Sigma-Aldrich) at a concentration of 1 mg/mL. They were then prepared on Middlebrook 7H10 agar medium supplemented with 10% (v/v) OADC and 0.5% (v/v) glycerol (Sigma) at a concentration of 0.1 mg/mL in the first step followed by a preparation with a final concentration of 0.01 mg/mL in a second step. These media were poured into 55-mm Petri dishes (Gosselin, Borre, France). Solubilization of chitin (Sigma-Aldrich) was achieved as previously described with few modifications by slowly dissolving chitin in 37% concentrated hydrochloric acid “HCl” (Sigma-Aldrich)^66^ (Supplementary material). The obtained colloidal chitin was then dissolved into Middlebrook 7H10 medium supplemented with 10% (v/v) OADC and 0.5% (v/v) glycerol at a concentration of 0.2 mg/mL and this medium was poured into 55-mm Petri dishes. The purity of chitin, fucose and NAG delivered by Sigma-Aldrich was controlled by MALDI-TOF-MS before incorporation into the enriched culture media. For each of the three reagents, one microliter of the supernatant was spotted per spot onto the polished-steel MSP 96 target plate (Bruker Daltonics) and allowed to dry at room temperature. Each dry spot was then overlaid with 1 μL of matrix solution (saturated α-cyano-4-hydroxycinnamic acid in 50% acetonitrile and 2.5% trifluoroacetic acid) (Bruker Daltonics). The plate was air-dried for 5 minutes and loaded for manual processing into the MALDI-TOF mass spectrometer (delay: 170 ns; ion source 1 (IS1) voltage: 20 kV; ion source 2 (IS2) voltage: 16.65 kV; lens voltage: 7.20 kV; mass range: 0 kDa to 1 kDa) taking into account the known molar mass of reagents. Molar mass was identified by the m/z (mass/charge) parameter on spectra. The specific peaks were obtained for chitin (molar mass of 627.59 g/mol), N-acetyl galactosamine (molar mass of 221. 20 g/mol) and fucose (molar mass of 164.15 g/mol) confirming the presence and purity of the reagents.

### Testing growth-promoters for *M. ulcerans* culture

For each one of the *M. ulcerans* strains under study, a 100 µL-volume of a 10^5^ AFB/mL suspension and PBS as negative control were cultured in parallel in triplicate onto each of the three enriched media and onto Middlebrooks 7H10 reference medium supplemented with 10% (v/v) OADC and 0.5% (v/v) glycerol. We seeded an inoculum of 10^4^ AFB by Petri dish rather that a greater concentration of AFB in order to minimize bias of counting because in bacterium kinetic, population growth strongly depends on initial conditions. The 55-mm plates were incubated at 30 °C in microaerophilic atmosphere. Five days, ten days, fifteen days, twenty days and thirty days after inoculation, colonies were observed by a MZ-FLIII fluorescence microscope (Leica, Nanterre, France) equipped with a GFP filter and an ICA digital camera (Leica) to detect mycobacterial autofluorescence^64^. Counting of fluorescent colonies was performed using the Leica Application Suite software (Leica)^64^. Identification of colonies was confirmed by MALDI-TOF-MS as previously described^65,67^ and by *rpoB* gene sequence analysis^63^.

### Decontamination of river freshwater experimentally inoculated with *M. ulcerans* strains

Freshwater obtained from the Huveaune River, a small river of southern France was used. In first time, we analyzed without decontamination formerly direct cultured samples of the river freshwater on 5% sheep-blood Columbia agar (COS, bioMérieux, La Balme-Les-Grottes, France) and after two days of culture we identified by MALDI-TOF-MS the growth bacterial colonies. The direct culture aimed to list bacteria and fungi contained in the native freshwater for the differential appreciation of germs between direct culture and culture after chlorhexidine decontamination. Secondarily, we decontaminated the freshwater of Huveaune River with 1% chlorhexidine and culture the pellet onto 5% sheep-blood Columbia agar. The decontamination was processed as previously described^68^.

The next step consisted of on the chlorhexidine decontamination of freshwater experimentally inoculated with *M. ulcerans* suspension. Briefly, in 50 mL Corning Falcon conical centrifuge tubes (Becton Dickinson) containing 5 mL of river freshwater experimentally inoculated with one milliliter of 10^7^ AFB/mL of each *M. ulcerans* Cu001 and *M. ulcerans* ATCC25900, the chlorhexidine decontamination was done as previously described^68^. Then, a 100-μL volume of pellet was inoculated in parallel on 5% sheep-blood Columbia agar, in triplicate on each of the growth-promoting media (chitin, N-acetyl galactosamine, fucose) and on the standard Middlebrook 7H10 medium. The negative control was consisted of seeding 100 µL of sterile PBS onto 5% sheep-blood Columbia agar, standard Middlebrook 7H10 medium and onto each of the growth-promoting media. Cultures and negative controls were examined at day 3, day 7 to appreciate the quality of the decontamination method and quality of the cultures. Colonies were counted at day 15 and day 30 using the Leica Application Suite software as described above.

### Isolation of *M. ulcerans* from environmental samples, Côte d’Ivoire

Tentative isolation of *M. ulcerans* was done on 12 water samples without plant debris (40–45 mL), 8 water samples with plant debris (40–45 mL) and 11 aulacode (*Thryonomys swinderianus*) feces collected around the Kossou dam near the village of Kongouanou, an endemic area of Buruli ulcer in the district of Yamoussoukro located in the centre of Côte d’Ivoire. The feces of aulacodes which are easy to recognize have been collected with the support of professional hunters around Kossou dam. All the samples were collected onto 50 mL Corning Falcon conical centrifuge tubes.

For the treatment of samples for seeding, water samples without and with plants debris were concentrated by centrifugation at 1,700 g for 15 min and the supernatants were removed. The resulting pellets from water without plant debris were suspended in PBS before decontamination. The resulting pellets from water with plant debris were suspended in PBS and mechanically disrupted with silica beads (0.5 mm diameter) and the obtained suspensions were transferred into 50 mL Corning Falcon conical centrifuge tubes before decontamination. 500 mg aulacode feces added into 50 mL Corning Falcon conical centrifuge tubes containing 5 mL of PBS were mechanically disrupted with silica beads (0.5 mm diameter) and the obtained suspensions were transferred into another 50 mL Corning Falcon conical centrifuge tubes before decontamination. Then, each sample was decontaminated using 1% chlorhexidine as previously described^68^. 100 µL of decontaminated pellet were seeded onto the chitin medium, NAG medium, fucose medium and DZ medium (Middlebrook 7H10 medium containing chitin (0.2%), NAG (0.01%) and fucose (0.01%)) and incubated for 24 weeks at 30 °C under a micro-aerophilic atmosphere condition using CampyGen Compact. 100 µL of sterile PBS was seeded in each media as negative controls and incubated in the same conditions. Colonies were screened with auto-fluorescence three times a month and the obtained colonies were identified by Real-Time PCR (RT-PCR) incorporating three independent gene targets, IS2404, IS2606 and KR-B, within the *M. ulcerans* genome^7,22,69,70^ and internal positive control to determine the level of inhibition as previously described^7^.

The presence of *M. ulcerans* DNA in environmental samples was analyzed by RT-PCR. We used the three primers (KR-B, IS*2404* and IS2606) to improve the specificity of the *M. ulcerans* DNA detection in the environmental samples^71,72^. Primers and probes from Applied Biosystems that were selected from regions of the sequences for IS2404, IS*2606* and KR present on the plasmid pMUM001 were used^7^. Probes IS2404TP and KR-BTP were labelled with the fluorescent dye 6-carboxyfluorescein (FAM) at the 5′ end and a non-fluorescent quencher at the 3′ end. Probe IS2606TP was labelled with the fluorescent dye VIC at the 5′ end and a non-fluorescent quencher at the 3′ end^7^ (Table 2). Total DNA was extracted from feces using the QIAmp® DNA Stool kit according to the manufacturer’s instructions (Qiagen, Stochach, Germany); total DNA was extracted from plant debris and sediment water using the NucleoSpin Tissue Kit (Macherey-Nagel, Hoerdt, France). *M*. *ulcerans* Cu001 DNA was extracted using a commercial Nucleospin Tissue kit (Macherey-Nagel, Hoerdt, France) which was used as a positive control in PCR-based identification of micro-colonies while distilled water was used as a negative control. The PCR inhibition was assessed by adding 10 μL of internal control into 190 μL of sample, as previously described^73^. Each IS2404*, IS*2606 *and KR* real-time PCR mixtures contained 5 μl of DNA or negative control, 20 μM of each primer, 5 μM of probe, 3.5 μL of sterile water and 10 μL of mastermix (Eurogentec) in a total volume of 20 μl. The RT-PCR program comprised one cycle at 50 °C for two minutes and 40 cycles at 95 °C for 15 seconds and 60 °C for one minute^22^, amplification was done in a CFX 96™real time PCR thermocycler and detection system (BIO-Rad, Marnes-la-Coquette, France). Two negative controls were incorporated into each PCR run. All samples were tested in triplicate. A specimen was considered as positive for the detection of *M*. *ulcerans* when both the insertion sequences IS*2606* and/or IS2404 and the KR-B detection were positive (Ct ≤ 40 cycles) in ≥2/3 replicates. The Ct cut-off value was chosen in order to increase the sensitivity of the detection as previously described^22^.

**Table 2:** Primers and probes designed for real-time PCR detection of *M. ulcerans* by targeting IS2404, IS*2606*, and KR-B gene.

Target sequence	Prime or Probe	N° of bases	Amplicon size	Sequences (5′–3′)	Nucleotide positions	No. of copies of amplicon per plasmid/chromosome
IS2404	IS2404 TF	19	59	AAAGCACCACGCAGCATCT	27746–27762	4/201
IS2404	IS2404 TR	18		AGCGACCCCAGTGGATTG	27787–27804	
IS*2404*	IS*2404* TP			6FAM-CGTCCAACGCGATC-MGBNFQ	27768–27781	
IS2606	IS2606 TF	21	58	CCGTCACAGACCAGGAAGAAG	28912–28932	8/82
IS2606	IS2606 TR	21		TGCTGACGGAGTTGAAAAACC	28947–28969	
IS2606	IS2606 TP			VIC-TGTCGGCCACGCCG-MGBNFQ	28933–28946	
KRB	KR-BTF	18	65	TCACGGCCTGCGATATCA	3178–3195	15/0
KR-B	KR-BTR	21		TTGTGTGGGCACTGAATTGAC	3222–3242	
KR-B	KR-BTP			6FAM-ACCCCGAAGCACTG-MGBNFQ	3199–3212	

### Statistics

The results of growth kinetics were expressed as mean value ± standard error of the mean (SEM) of counted colonies. Analysis of variance (ANOVA) was used for statistical analysis. P values lower than 0.05 (p < 0.05) were considered statistically significant. The doubling time was determined by calculating the average slope of the mycobacterial replication curve during the exponential phase of culture using the following formula: growing ratio: µx = lnxB-lnxA/tB-tA and doubling time: G = ln2/µx.

## Electronic supplementary material

Supplement 1
