# Supplementary material for: A protocol for culturing environmental strains of the Buruli ulcer agent, Mycobacterium ulcerans
Source: Sci Rep. 2018 Apr 30;8:6778. doi: 10.1038/s41598-018-25278-y (PMC5928104; doi:10.1038/s41598-018-25278-y)
Supplement: Supplementary file 1 — Supplement 1 [file 41598_2018_25278_MOESM1_ESM.pdf]

A protocol for culturing environmental strains of the Buruli ulcer agent, *Mycobacterium ulcerans*

Dezemon Zingue[1], Arup Panda[1, 2], Michel Drancourt[1*]

Supplement 1: Supplementary information

Chitin dissolution protocol

Ten grams of chitin were slowly dissolved in 400 ml of 37% concentrated HCl in a 1000 ml beaker. The HCl was added slowly with continuous stirring with the use of a glass pipette for 5 minutes, followed by stirring for 1 minute at an interval of every 5 minutes for 60 minutes in a chemical fume hood at room temperature (25 ° C). The mixture was incubated in a water bath at 37°C until viscosity decreased. The chitin- HCl mixture was then passed through 8 layers of cheesecloth to remove large chitin chunks. The clear filtrate obtained (100 ml) was then treated with 2 liters of ice cold sterile distilled water to allow precipitation of colloidal chitin. This was incubated overnight under static conditions at 4°C to facilitate better precipitation of colloidal chitin. This was later passed through two layers of coffee filter paper, housed in a Buchner funnel (130 mm) seated in a vacuum filtration flask under vacuum. Approximately 3 liters of tap water (pH of ~8.0) were passed through the colloidal chitin cake using this filter assembly, until the pH of the filtrate had risen to 7.0 (estimated by pH paper). The colloidal chitin obtained was pressed between coffee filter papers (to remove additional moisture), and then placed in a 100 ml glass beaker covered with two layers of aluminum foil and sterilized by autoclaving at standard temperature and pressure (STP) (15 psi, 20 minutes, 121 ° C). The autoclaved colloidal chitin was stored at 4°C until further use. The colloidal chitin we obtained had a soft cake-like texture.
